# Right ventricular function and dimensions in type 2 diabetes mellitus

**DOI:** 10.1186/1532-429X-14-S1-P71

**Published:** 2012-02-01

**Authors:** Ralph Widya, RW Van Der Meer, Johannes W Smit, Luuk Rijzewijk, Michaela Diamant, Jeroen J Bax, Albert de Roos, Hildo J Lamb

**Affiliations:** 1 Radiology, Leiden University Medical Center, Leiden, Netherlands; 2 Endocrinology, Leiden University Medical Center, Leiden, Netherlands; 3 Diabetes Center, Department of Internal Medicine, VU University Medical Center, Amsterdam, Netherlands; 4 Cardiology, Leiden University Medical Center, Leiden, Netherlands

## Background

Right ventricular (RV) function has proven to be of importance for patient risk stratification in heart failure and is associated with sudden death and exercise limitation. RV dysfunction might play an important role in diabetic cardiomyopathy. We therefore assessed RV function and volumes using MRI in patients with otherwise uncomplicated type 2 diabetes mellitus (T2DM), and compared RV parameters with those of the left ventricle (LV).

## Methods

Steady-state free precession sequences were used to assess RV dimensions in 78 uncomplicated T2DM patients and 28 healthy subjects within the same range of age. Furthermore, flow velocity mappings across the pulmonary valve and tricuspid valve were used to assess RV outflow and diastolic filling patterns respectively.

## Results

RV end-diastolic volume was significantly decreased in patients compared to healthy subjects, also after adjustment for BMI and pulse pressure (177 ± 28 ml vs. 197 ± 47 ml, P<0.01). RV systolic function was impaired: Peak ejection rate across the pulmonary valve was significantly lower (433 ± 54 vs. 463 ± 71 mL/s, P<0.01). Indexes of RV diastolic function were impaired: E peak filling rate and E deceleration peak were 315 ± 63 ml/s vs. 356 ± 90 ml/s, P<0.01 and 2.3 ± 0.8 ml/s^2^ * 10^-3^ vs. 2.8 ± 0.8 mL/s^2^ * 10^-3^, P<0.01, respectively. All RV parameters strongly correlated with its corresponding LV parameter (P<0.001).

## Conclusions

Diabetic cardiomyopathy affects the right ventricle, similar to changes in LV dimensions and function. These observations suggest that right ventricular impairment is a component of the diabetic cardiomyopathy phenotype.

## Funding

This research was performed within the framework of the Center for Translational Molecular Medicine (www.ctmm.nl), project PREDICCt (grant 01C-104), and supported by the Netherlands Heart Foundation, the Dutch Diabetes Research Foundation, the Dutch Kidney Foundation, and Eli Lilly, the Netherlands.

**Figure 1:**
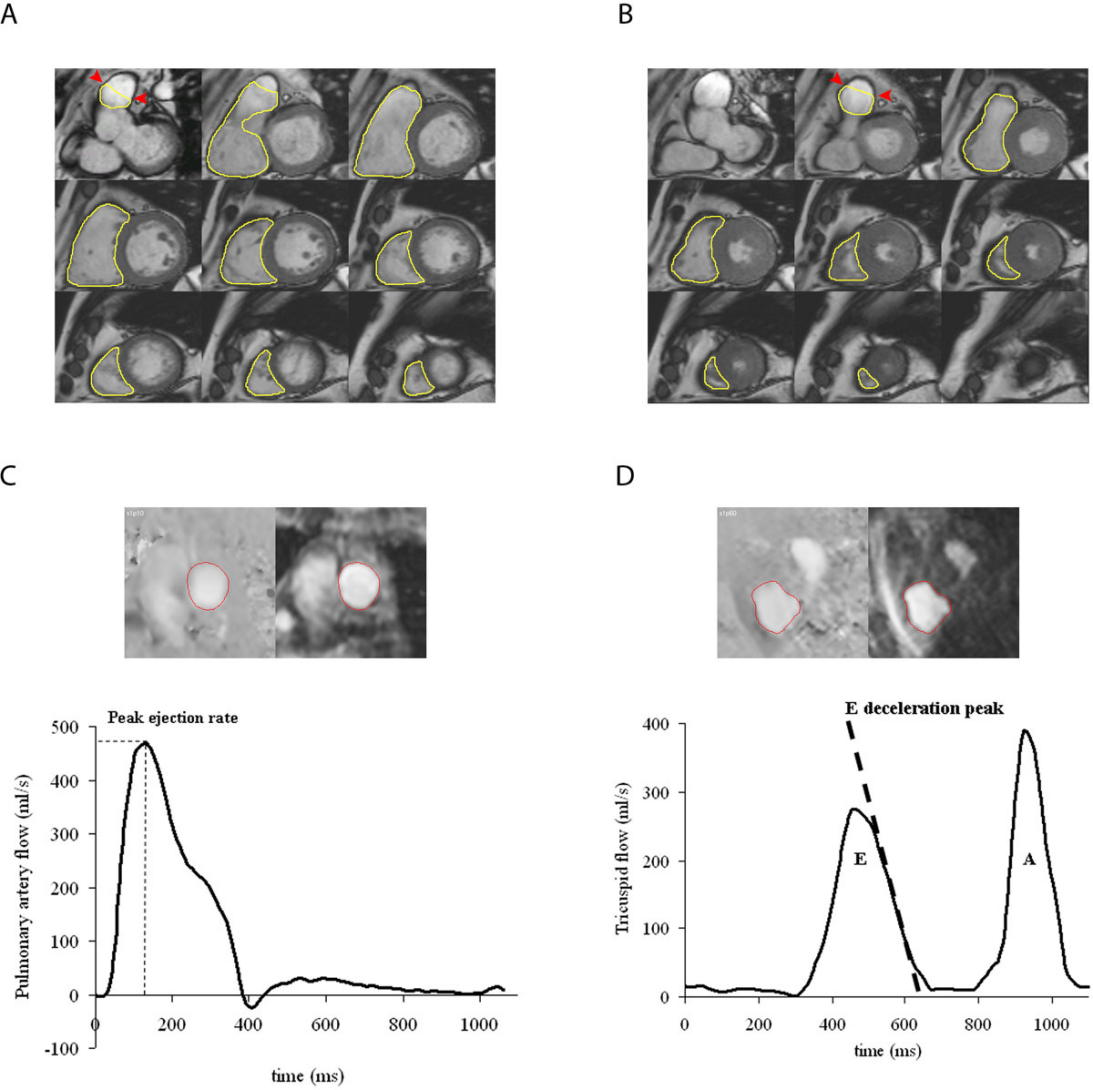
Example of MRI analyses of the right ventricle (RV). Endocardial contours of the RV in end-diastolic phase (A) and end-systolic phase (B) are manually drawn on the short-axis view to assess volumes and RV systolic function. The papillary muscles, trabeculae carnae, trabecula septomarginalis (moderator band), and RV outflow tract are included in the ventricular volume. The RV outflow tract ends at the pulmonary valves (arrowheads). Furthermore, pulmonary artery flow curve is obtained. A phase contrast velocity map across the pulmonary valve (left) and a modulus image (right) are shown in one cardiac phase (C). RV inflow curve across the tricuspid valve was used to assess RV diastolic function. A phase contrast velocity map across the tricuspid valve (left) and a modulus image (right) are shown in one cardiac phase (D). E indicates early diastolic filling phase; A, atrial contraction.

**Table 1:** Magnetic resonance study parameters.

	Healthy subjects n = 28	T2DM patients n = 78
RV dimensions		

End-diastolic volume (mL)	197 ± 47	177 ± 28 *†
End-systolic volume (mL)	93 ± 28	83 ± 18 *†
End-diastolic volume index (mL * m^-2^)	95 ± 20	84 ± 12 *†
End-systolic volume index (mL * m^-2^)	45 ± 12	39 ± 8 *†

RV systolic function		

Stroke volume (mL)	104 ± 21	95 ± 15 *†
Stroke volume index (mL * m^-2^)	50 ± 9	45 ± 6 *†
Cardiac output (mL/min)	6060 (5432-6661)	6227 (5524-7091)
Cardiac index (L x min^-1^ * m^-2^)	3.0 (2.7-3.2)	2.9 (2.6-3.2)
Ejection fraction (%)	53 ± 4	54 ± 5
Peak ejection rate (mL/s)	463 ± 71	433 ± 54 *†

RV diastolic function		

E peak filling rate (mL/s)	356 ± 90	315 ± 63 *†
E deceleration peak (mL/s^2^ * 10^-3^)	2.8 ± 0.8	2.3 ± 0.8 *†
A peak filling rate (mL/s)	349 ± 60	353 ± 68
E/A	0.95 (0.82-1.28)	0.85 (0.73-1.06) *

Data are mean ± SD or median (interquartile range); * P<0.05 uncorrected for pulse pressure, and BMI when appropriate; † P<0.05 after correction for pulse pressure, and BMI when appropriate.

